# Genomic and proteomic analyses of the maize root isolate *Rhodococcus erythropolis* NI86/21 reveal extensive genome plasticity and parallel evolution of herbicide degradation

**DOI:** 10.1128/aem.02407-25

**Published:** 2026-01-28

**Authors:** Judit Kosztik, Erzsébet Baka, András Táncsics, Renáta Ábrahám, Gyula Szabó, István Nagy, Massimiliano Orsini, Ildikó Bata-Vidács, Helga Szalontai, József Kukolya, István Nagy

**Affiliations:** 1 Department of Research and Development, Eszterházy Károly Catholic University 72314, Eger, Hungary; 2 Department of Molecular Ecology, Institute of Aquaculture and Environmental Safety, Hungarian University of Agriculture and Life Sciences 72402, Gödöllő, Hungary; 3 Department of Environmental Toxicology, Institute of Aquaculture and Environmental Safety, Hungarian University of Agriculture and Life Sciences 72402, Gödöllő, Hungary; 4 Seqomics Biotechnology Ltd., Mórahalom, Hungary; 5 Biological Research Centre, HUN-REN, Institute of Biochemistry 211214 , Szeged, Hungary; 6 Laboratory of Microbial Ecology and Genomics, Istituto Zooprofilattico Sperimentale delle Venezie, Legnaro, Italy; University of Milano-Bicocca, Milan, Italy

**Keywords:** *Rhodococcus erythropolis *NI86/21, genome plasticity, proteomics, horizontal gene transfer, herbicide degradation

## Abstract

*Rhodococcus erythropolis* NI86/21, isolated from maize rhizosphere in Hungary, possesses one of the largest genomes (8.046 Mb) within the species. The genome comprises a 6.83 Mb chromosome and 1.22 Mb of extrachromosomal elements, including three circular and two fragmented linear plasmids. Comparative analysis identified five horizontally acquired genomic islands (HGTi), totaling 0.64 Mb with mosaic-like architecture derived from plasmids, phages, and chromosomal segments of other *Nocardiaceae*. Liquid chromatography-tandem mass spectrometry (LC-MS/MS)-based proteomic analysis revealed a lower expression of genes located in HGT elements (53%) compared to core chromosomal genes (73%), indicating regulatory silencing of foreign DNA. Nevertheless, an inducible cytochrome P450 monooxygenase (CYP116) responsible for thiocarbamate and atrazine degradation is encoded on HGTi_V and actively expressed upon herbicide exposure. Strikingly, an identical CYP450 locus is present on a conjugative plasmid in *Rhodococcus* sp. TE1 isolated from thiocarbamate-treated soil in Canada, demonstrating independent acquisition of the same catabolic module from a high GC% content *Rhodococcus*, under similar selective pressure. Frequent recombination between chromosomal and mobile elements generates the observed mosaic-like HGT structures, which we found common for *R. erythropolis* strains. These results highlight extraordinary genomic plasticity and rapid adaptive evolution in *Rhodococci*, enabling efficient colonization of herbicide-contaminated agro-ecosystems.

**IMPORTANCE:**

*Rhodococcus erythropolis* NI86/21 exemplifies how bacterial genomes evolve through horizontal gene transfer and mobile elements. Its unusually large, plastic genome contains extensive HGT islands and a high load of active transposons, which shape mosaic genomic architectures and hinder complete genome assembly. These horizontally acquired regions, although partially silenced, encode key adaptive functions such as an inducible CYP116 monooxygenase enabling thiocarbamate and atrazine degradation. Remarkably, an identical CYP116 module is present in *Rhodococcus* sp. TE1 from thiocarbamate-treated Canadian soil, demonstrating that similar environmental pressures can drive independent acquisition of the same biodegradation trait. Together, the dynamic transposon activity, mosaic HGT structure, and geographically convergent gene recruitment highlight the extraordinary genomic plasticity of *R. erythropolis* and underscore its rapid adaptive potential in agro-ecosystems, with implications for microbial evolution and bioremediation strategies.

## INTRODUCTION

Rhodococci are Gram-positive, high GC% content, aerobic, nocardioform actinomycetes inhabiting highly diverse niches. Members of the genus *Rhodococcus* are regarded as masters of biodegradation, as they can transform and degrade a wide range of xenobiotic compounds (1). Due to their relevance in environmental and industrial microbiology and biotechnology, the genome sequences of hundreds of isolates have been elucidated. Many of the enzymes responsible for xenobiotic degradation and heavy metal resistance have been found encoded on mobile genetic elements (1), and their acquisition and maintenance in the cell are attributed to horizontal gene transfer (HGT) and genome plasticity (2). The genus *Rhodococcus* belongs to the family Nocardiaceae and is part of the wider *R. erythropolis*/*R. qingshengii* species complex, whose members exhibit extensive genomic diversity. Comparative genomic studies have shown that *Rhodococcus* possesses an open pan-genome, driven by frequent gene gain and loss, large accessory genome fractions, and widespread plasmid carriage. Genome sizes typically range from 5.5 to 7.5 Mb across the genus, with occasional expansions beyond 8 Mb in strains with extensive horizontally acquired DNA. This variability, combined with a high GC% content and a modular chromosomal architecture, reflects the ecological versatility characteristic of the genus (3). Genome plasticity allows organisms to adapt to environmental changes and occupy novel niches. It is an interplay between gene loss and gene gain/acquisition, while the bacterial cells have to be protected against undesired mobile genetic elements and invading phages and have to maintain a stable genome. Conjugative transfer of bacterial plasmids is the most efficient way of horizontal gene spread (4). The process relies on surface adhesins for mediating contact between donor and recipient cells, and a plasmid’s mobility (MOB) region, containing genes for a relaxase (or nickase) and a type IV secretion system (T4SS), which cleave the DNA at the origin of transfer (oriT) and facilitate its transfer to the recipient cell (4). Genes acquired by HGT can be expressed and provide a fitness benefit to the recipient cell by effectively interacting with the recipient proteins (5). HGT-derived genes form part of the accessory genome, distinct from the core genome and other chromosomal genes characteristic of the species. Conjugative plasmid transfer has been described in a variety of actinobacteria, including *Rhodococcus* (6). A chromosome with an average size of around 6.5 Mb and the presence of linear and circular plasmids with variable length and copy numbers are characteristic of *R. erythropolis* isolates. *Rhodococcus* linear plasmids have a composite structure (7), comprising a conserved backbone with genes for plasmid maintenance and transfer, and a variable region with horizontally acquired genes that provide niche-specific functions. Actinobacterial linear replicons usually show invertron-type telomeres, which are proposed to have evolved from bacteriophages (8). Linear plasmids in *Rhodococcus* and related actinobacteria are of particular functional importance, as they frequently encode catabolic pathways, stress response systems, and adaptive metabolic modules that are absent from the chromosome. These replicons can exceed several hundred kilobases in size and often contribute disproportionately to strain-specific metabolic versatility, including the degradation of xenobiotics and other niche-defining traits. Although circular plasmids are more widely distributed across *R. erythropolis*, large linear plasmids are increasingly recognized as major drivers of genome expansion and ecological specialization within the genus (9).

Besides plasmids, HGT islands—substantial segments of foreign DNA embedded within bacterial genomes—also can increase the genome size. They can have distinct nucleotide composition reflected by divergent GC% content and—just like plasmids—frequently harbor clusters of genes associated with specialized functions. *R. erythropolis* strains do not have well-documented chromosomal HGT islands in their chromosome, but it has been shown that rhodococci possess mechanisms for high-frequency and illegitimate recombination (10). Studies of plasmid integration in *R. fascians* (11) have shown the involvement of a short palindromic sequence (CCGCGG) and that exogenous DNA, bearing such a sequence, could lead to a non-homologous integration at a recombinational “hot-spot” sequence. Although this phenomenon has not been extensively studied, it is possible that rhodococci recombine easily with many heterologous sequences (12).

Transposable genetic elements (TGE) are segments of DNA that can move within a genome and be transferred between different organisms. These elements play a significant role in evolution by genome change. TGEs include transposons and insertion elements, and it is widely assumed that TGEs, which do not have enzymatic machinery for transfer between host cells, use plasmids and phages as cargo for HGT (13). *R. erythropolis* TGEs are well documented, and their use in molecular biology has great industrial benefits (14). One of the best examples for the expression and activity of rhodococcal transposases is the KO mutagenesis of the *par*A and *par*B genes of *R. erythropolis* PR4 using the Flp-*FRT* site-directed recombination technology. This system uses unspecified endogenous recombinases for the desired recombination/KO mutagenesis event (15).

To maintain genome stability, prokaryotes have developed different strategies. Toxin-antitoxin (TA) loci are small genetic modules commonly found in their chromosomes and plasmids (1). Typically, a TA locus consists of a pair of genes: one encodes a stable toxin that inhibits essential cellular functions, and another encodes a labile antitoxin that neutralizes the activity of its cognate toxin. TA systems have been found to participate in various biological processes essential for bacterial survival and adaptation, such as stress response, bacteriophage defense, bacterial virulence, antimicrobial resistance, and plasmid stability (16). The known TA loci can be classified into eight types, I–VIII (17, 18). Most described toxins are proteins (types I–VII), while the antitoxins could be proteins or RNA. Growing evidence indicates significant associations between TA loci and mobile genetic elements, highlighting their functional interplay (19–21).

Bacterial populations are under constant threat from bacteriophages, which represent one of the primary causes of bacterial mortality. This relentless evolutionary pressure, shaped by billions of years of host-phage coevolution, has driven bacteria to evolve sophisticated defense mechanisms to detect, prevent, and neutralize phage infections (22). The effectiveness of these defense systems is crucial for bacterial survival, population stability, and ecological success.

One of the first *Rhodococcus* strains reported to utilize/degrade xenobiotics was *R. erythropolis* NI86/21 strain (*Arthrobacter* sp. NI86/21 or *Rhodococcus* sp. NI86/21 or NI1), which we isolated from an S-ethyl dipropylcarbamothioate (EPTC) herbicide-treated soil in Hungary as a thiocarbamate and triazin herbicide degrader (23). As a result of the research work aiming to find genes/gene products responsible for this activity, four upregulated proteins were identified by two-dimensional polyacrylamide gel electrophoresis (2D-PAGE)/Edman degradation-based proteome analysis. The ThcB protein (a CYP450 clustered with an electron transfer chain consisting of a ferredoxin and a ferredoxin reductase) was responsible for the initiation of N-dealkylation of thiocarbamate herbicides (EPTC, butylate, vernolate, and cycloate) (24, 25). Two other identified proteins, an aldehyde dehydrogenase (ThcA) and an N,N-dimethyl-4-nitrosoaniline (NDMA)-dependent methanol dehydrogenase (ThcE), are responsible for metabolizing the cleaved alkyl side chains (26). The fourth protein is a non-heme chloroperoxidase (ThcF), whose role is not fully understood in thiocarbamate degradation (27).

We found a truncated copy of the 20S proteasome operon upstream of the *thc*B gene cluster, which prompted us to isolate and characterize the first eubacterial 20S proteasome (18). The quaternary structure of the 20S catalytic core particle of 20S and 26S proteasomes of prokaryotes and eukaryotes, respectively, resembles a barrel built from four rings. The two outer rings consist of seven α subunits, and the two inner rings consist of seven β subunits. The proteolytic active sites are situated on the β subunits, and they are positioned in the inner cavity formed by β rings. They are activated during the assembly process of the 20S particles by autocatalytic self-cleavage of the β pro-peptide. By this process, the catalytic active site threonine residue remains hidden until the 20S particle is fully assembled, avoiding non-targeted proteolysis. The outer α rings allow the docking of the hexameric ATPase ring for the ATP-dependent unfolding of ubiquitin-labeled (eukaryotes) or PUPylated (actinomycetes) proteins destined for targeted proteolysis (28). In prokaryotic 20S particles, there is only one copy of α and one copy of β subunits, while eukaryotic 20S particles are built from seven copies of α and seven copies of β subunits. The isolated NI86/21 20S particles showed an intermediate complexity, as they consist of two α and two β subunits. Subsequent searches elucidated the presence of the complete 20S operon as well, indicating that the genes of the truncated 20S operon were obtained by HGT.

A copy of the IS1415 insertion element was found downstream of the CYP450 gene cluster, and later, southern hybridization experiments found three copies of it. By inserting a chloramphenicol-resistant gene in the IS element, we developed a transposon (pFAJ2571), which was successfully integrated into the genome of several *Rhodococcus* strains (29), providing a useful tool for generating mutant libraries. This transposon was further developed, producing an industrially relevant construct, which could jump into the chromosome of rhodococci in several positions, multiplying the expression level of recombinant proteins (19).

Based on isolation and sequencing of the 6 kb cryptic plasmid of the NI86/21 strain, another useful genetic tool, the *Rhodococcus-E. coli* shuttle vector (pFAJ2574) was developed. This plasmid vector demonstrates stable maintenance across multiple *Rhodococcus* strains, both in the presence and absence of the selective agent chloramphenicol (20).

*R. erythtropolis* strains have been demonstrated to be generally good mycotoxin degraders, and the NI86/21 (NI1) strain proved to be one of the best in terms of completeness of degradation process on investigated mycotoxins (30). One of our near-future objectives is to identify the genes and gene products responsible for aflatoxin B1 degradation. To achieve this, genome sequencing and annotation are crucial, which prompted us to sequence the genome of the NI86/21 strain. Despite extensive genomic data for the genus, little is known about the expression patterns of horizontally acquired genes in *Rhodococcus* spp. or the relationship between plasmid-encoded and chromosomal gene expression under optimal laboratory growth conditions.

After sequencing and annotating the NI86/21 genome, it was obvious that it has the largest genome and chromosome among reliably sequenced *R. erythropolis* strains, derived by acquiring three circular and two linear plasmids, and five HGT islands in the chromosome. These findings prompted us to analyze the genome deeper with bioinformatic tools and the proteome by LC-MS/MS experiments to reveal and compare the protein expression rate of the core and the accessory genomes. Besides, we were interested in identifying the origin of the genomic environment of the truncated copy of the proteasomal operon and the *thc*B and *thc*F genes, which conferred unique properties to the NI86/21 strain.

## MATERIALS AND METHODS

### Growth conditions and tDNA extraction

For culturing *R. erythropolis* NI86/21 and treating the cells with the cell wall inhibitor ampicillin to weaken the cell wall for tDNA extraction, we used the identical protocol we described previously (24). Briefly, cells were grown in 200 mL Luria-Bertani broth at 28°C until cell density reached OD_600_ of 0.7–1.0, then ampicillin was added at 600 µg/mL final concentration, and the cells were incubated further for 4 h. Cells were centrifuged, washed, and resuspended in 10 mL Tris/EDTA 10/1 pH 8.0 buffer (TE buffer). Lysozyme was added at 2 mg/mL final concentration, and the suspension was incubated for 1 h at 37°C, after which the incubation was continued in the presence of 0.75 mg Pronase at 55°C for 30 min. After this, 2 mL of 5 M NaCl and 10 mL of 1% SDS were added, and the cell lysate was gently shaken until complete cell lysis. After this, RNase was added, and the lysate was incubated for 10 min at room temperature (RT), after which one volume of 24:1 chloroform:isoamyl alcohol was added, and the genetic material was extracted by gentle shaking at RT for 30 min. The lysate was centrifuged at 13,000 rpm for 30 min, and the supernatant was mixed with two volumes of ethanol (EtOH). The precipitated DNA was washed three times with 70% EtOH, and after drying, it was dissolved in 5 mL TE buffer. The lysate was kept at 4°C to avoid DNA damage caused by freezing and thawing.

### Genome sequencing and assembly

The genome of NI86/21 was sequenced using Pacific Biosciences (PacBio) and Illumina technologies. PacBio sequencing library was constructed and subsequently sequenced on the PacBio Sequel platform, according to the manufacturer’s instructions. The Illumina sequencing library was constructed and sequenced using the Illumina MiSeq platform as described previously (31). Assembly, using the PacBio reads only, was then carried out using Ra (v0.2.1), yielding 16 contigs; subsequently, Illumina reads were aligned to the PacBio assembly using CLC Genomics Workbench (v11.0.1), with an average coverage of 331×. Annotation of the genome was performed by NCBI Prokaryotic Genome Annotation Pipeline v4.11 with Best-placed reference protein set and GeneMarkS 2+ methods (32, 33).

### Functional annotation

Functional annotation of the translated coding sequences was conducted using the NCBI CD-Search tool (https://www.ncbi.nlm.nih.gov/Structure/bwrpsb/bwrpsb.cgi) to identify conserved domains by using the COG (Clusters of Orthologous Groups of Proteins) database (34) under default parameters. Because some proteins were assigned to multiple COG categories, the relative abundances of functional categories were normalized by dividing the number of hits per category by the total number of assigned functional domains. Mauve v.2.4.1 software was used for multiple sequence alignment (35). The functional annotation of coding sequences was conducted using the DIAMOND sequence aligner tool (36). The Orthovenn3 platform was used to identify orthologous clusters (37). The COG analysis was conducted using the COG classifier v.1.0.5 (38). Prophage regions in the genome were identified using multiple complementary tools, including Phigaro, VirSorter, and PHASTER embedded into the Proksee bioinformatics platform to ensure robust detection and cross-validation of prophage sequences (39–42). In cases where the usage of bioinformatic platforms provided no results for obvious hits, we filtered the annotated genes by protein names provided by the annotation platform to get information on number of genes belonging to categories of interest. To identify and characterize plasmid sequences within the genome of NI86/21, we employed PlasmidHunter (43).

### Protein extraction and preparation for LC-MS/MS analysis

#### Sample preparation

NI86/21 cells were grown in 50 mL LB medium in 250 mL flasks at 28°C in a rotary shaker for 2 days at 180 rpm until they reached late log phase. Cells of five parallel samples were collected and washed with bidistilled H_2_O, and cell pellets were stored at −80°C until usage. The cell pellets were incubated with 0.4 mL of preheated SDC buffer containing 1% sodium deoxycholate (SDC, Sigma-Aldrich), 40 mM 2-chloroacetamide (CAA, Sigma-Aldrich), 10 mM tris(2-carboxyethyl)phosphine (TCEP; Thermo Fisher Scientific), and 100 mM Tris, pH 8.0. After incubation for 5 min at 95°C, the samples were ultrasonicated for 2 min with 0.5 s pulses (50% intensity) and 2 s pauses (Sonopuls, Bandelin). Incubation for 5 min at 95°C and subsequent ultrasonication were repeated. The samples were diluted 1:1 with MS-grade water (VWR). Proteins were digested by incubation with 1 µg Lys-C (Wako) for 4 h and then incubated overnight at 37°C with 1 µg of trypsin (Promega). The peptide solution was then acidified with trifluoroacetic acid (Merck) to a final concentration of 1%, followed by desalting via SCX stage-tips.

### LC-MS/MS data acquisition

Peptides were eluted from the Evotips onto a 15 cm PepSep C18 column (1.5 µm, Bruker Daltonics) using the Evosep One HPLC system (Evosep). The column was maintained at 50°C, and peptide separation was achieved using the 30 SPD method. Eluted peptides were directly ionized and introduced into a trapped ion mobility time of flight (timsTOF) Pro mass spectrometer (Bruker) via electrospray ionization. Data acquisition was performed in data-independent acquisition (DIA) PASEF mode via timsControl. Mass spectrometry covered a scan range of 100–1,700 m/z, and ion mobility ranged from 1/K0 = 0.70 to 1.30 Vs·cm². The dual TIMS analyzer utilized equal ion accumulation and ramp times of 100 ms each, with a spectral rate of 9.52 Hz. For DIA-PASEF scans, the mass scan range was 350.2–1,199.9 Da, and ion mobility ranged from 1/K0 = 0.70 to 1.30 Vs·cm². Collision energy was linearly ramped based on ion mobility, from 45 eV at 1/K0 = 1.30 Vs·cm² to 27 eV at 1/K0 = 0.85 Vs·cm². A total of 42 DIA-PASEF windows were acquired per TIMS scan, with alternating precursor isolation windows, resulting in an estimated cycle time of 2.21 s.

### Data analysis

Raw data were analyzed using Spectronaut (v19, Biognosys) in directDIA+ (library-free) mode. The data were searched against a predicted library of *R. erythropolis* NI86/21 with standard settings applied. Cysteine carbamidomethylation was set as a static modification, and methionine oxidation and N-terminal acetylation were set as variable modifications. The protease was set to Trypsin/P.

## RESULTS

The genome of *R. erythropolis* NI86/21 was sequenced using a hybrid approach combining Illumina short-read and PacBio long-read sequencing technologies. The used tDNA extraction method provided a high-quality sample, since even the broken chromosomal DNA fragments were bigger than 50 kb, and both circular and linear megaplasmids could be detected in unfrozen samples (Fig. S1). The PacBio sequencing yielded 1.787 Gb of subread bases across 202,207 subreads, with an average subread length of 8,836 bp and an N50 of 10,044 bp, providing long-range information essential for resolving repetitive regions. Despite the high DNA quality, we could not achieve full genome assembly. The assembly yielded a genome size of 8.046 Mb, organized into 16 contigs. This genome size significantly exceeds the average size reported for *R. erythropolis* strains, which typically ranges from 6.2 to 7.1 Mb. The substantial variation in total genome size across strains is largely attributable to differences in the number and composition of extrachromosomal elements, including both linear and circular plasmids. Notably, only two strains—NI86/21 and NPDC097342—exhibit genome sizes greater than 8 Mb (8.046 and 8.272 Mb, respectively), but the latter one is sequenced only to the scaffold level (848) with 8.109 annotated proteins, raising doubts about the reported sequence length. Among the fully assembled *R. erythropolis* genomes, the average chromosomal size lies between 6.2 and 6.5 Mb. The genome annotation of NI86/21 identified 7,657 genes, of which 7,483 were protein encoding, which number decreased to 7,144 after removing multiple copies of genes. The genome of *R. erythropolis* NI86/21 contains a full complement of ribosomal RNA operons (five each of 5S, 16S, and 23S rRNAs), 58 tRNAs, three noncoding RNAs (ncRNAs), and 95 pseudogenes corresponding to coding sequences lacking predicted protein products. The PlasmidHunter platform categorized the 4.7 and 2.09 Mb contigs as chromosomal and the smaller contigs as plasmid DNA (Table S1 **-** PlasmidHunter). Contigs 6, 7, and 16, with 106,991 bp, 106,078 bp, and 5.9 kb sizes, respectively, are fully assembled circular plasmids. The rest of the contigs show high similarity to stretches of linear plasmids (11 contigs with 999.023 bp), and they belong to two linear plasmids (Fig. S1). These plasmids encode for telomere binding and terminal proteins (RS33050, RS33055, RS37050, and RS37055, respectively) close to the 3′ ends of Contigs 3 and 9. To divide the contigs between the two plasmids was not possible, as their sequences were mosaic-like, giving homologies to different, mostly *R. erythropolis* and *R. qingshengi*, plasmids. Parts of Contigs 5, 14, and 15 showed high similarity to linear plasmids and chromosomal DNA fragments, further emphasizing their mosaic-like architecture.

### Comparative genome alignment, and virulence and resistance factors of the NI86/21 strain

**Fig 1:**
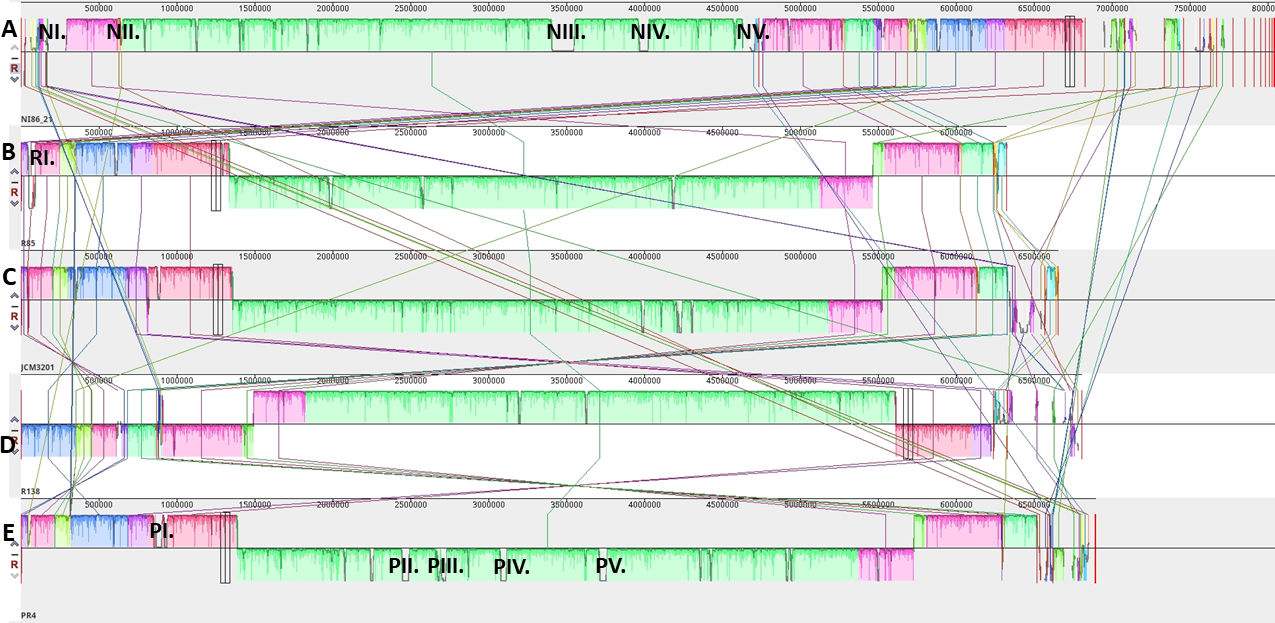
Whole-genome Mauve alignment of R. erythropolis NI86/21 (**A**) with strains R85 (**B**), JCM3201 (**C**), R138 (**D**), and PR4 (**E**). Colored blocks indicate chromosomal contigs; gaps indicate extrachromosomal or nonhomologous regions. Roman numerals mark HGT islands (NI–NV in NI86/21, PI–PV in PR4, RI in R85).

The complete genome sequence of NI86/21 was subjected to comparative alignment against four fully sequenced *R. erythropolis* reference strains—R85, JCM3201, R138, and PR4—using the Mauve genome alignment platform. This analysis revealed five distinct regions of unaligned gaps, within the primary 4.7 Mb contig of NI86/21, designated as HGTi I–V, located at the following genomic coordinates: I (1–280,743 bp), II (616,550–652,045 bp), III (3,402,921–3,549,924 bp), IV (3,960,001–4,029,333 bp), and V (4,627,077–4,735,238 bp) (Fig. 1). HGT-derived sequences are present on the ends of the chromosomal contigs and three of the ends (the 3′ end of C1 and both ends of C2) are terminated by partial sequence of the novel 19 kb Tn7706 transposon.

Subsequent BLASTN analyses facilitated classification of the HGTi into four distinct categories based on sequence similarity: (i) linear plasmid-derived sequences, (ii) chromosomal DNA segments originating from phylogenetically related, but distinct bacterial species, (iii) prophage-associated elements, and (iv) genomic fragments lacking any similarities in current searchable databases. Notably, these HGTi displayed characteristic composite architecture (Table S2), indicative of multiple recombination events and horizontal gene acquisition.

The 281 kb HGTi_I island exhibited a mosaic-like organization, containing both unique and homologous regions. Segments spanning 0–60 kb, 185–215 kb, and 235–252 kb showed no detectable similarity to known sequences, whereas the intervening regions (60–185 kb, 215–235 kb, and 252–281 kb) displayed homology to *R. erythropolis* XP plasmid pXP3, *R. erythropolis* DN22 plasmid pL2, and *R. qingshengii* DC2 plasmid unnamed2, respectively. Notably, a 15-kb stretch within plasmid pXP3 (85–100 kb) contained a chromosomal fragment of *Streptomyces* origin, sharing high identity with sequences from *Streptomyces* sp. NBC_00334, *Streptomyces mutabilis*, and *Rhodococcus hoagii* JCM9425, as well as with the unnamed plasmid2 of *Streptomyces* sp. NBC_00334. This region encodes several proteins, including the ThcF (RS00470) enzyme ribosome.

The HGTi_II showed sequence similarity to *Rhodococcus* sp. 008 plasmid pRC1, while HGTi_IV represented a chromosomal “no match” region characterized by five conserved ~1-kb segments exhibiting similarity to *R. erythropolis* and *R. qingshengii* chromosomes, spaced approximately 10 kb apart. Functionally, this region encodes biosynthetic genes for nonribosomal peptide synthetase (NRPS) and polyketide synthase (PKS) enzymes, suggesting potential involvement in secondary metabolite production.

HGTi_III was composed of two distinct regions: a 0–80 kb segment lacking similarity to known sequences (“no match”), followed by an 80–148 kb region displaying prophage-related features. BLASTN analysis of the 114.9 kb HGTi_V island revealed a compartmentalized structure. The first 0–55 kb segment had no significant homology to known sequences, while the 55–82 kb region aligned with *Rhodococcus* chromosomal DNA and included a truncated proteasome operon typically found on actinobacterial chromosomes. The 82–115 kb segment shared sequence identity with *Gordonia sihwensis* B403 chromosomal DNA and the pRhX5 plasmid of *R. erythropolis* X5, and the final ~1-kb fragment corresponded to a Tn7706 transposon sequence, indicating the presence of mobile genetic elements.

Analysis of plasmid contigs further supported a mosaic-like structure. Contigs C3 and C4 contained large DNA regions of approximately 70 kb and 160 kb (gene clusters RS31875–RS32250 and RS33445–RS34420, respectively), showing high sequence similarity to linear plasmids from *Mycolicibacterium frederiksbergense* strain LB501ᵀ (plasmid unnamed1) and *Mycobacterium* sp*.* MS1601 (plasmid unnamed). Smaller contigs resembled fragments of linear plasmids or exhibited similarity to *R. erythropolis* chromosomal and plasmid DNA.

A search for plasmid-encoded conjugal transfer proteins (CTPs) identified several type II/IV secretion system components, including ATPases, flagellar and pilus assembly proteins, and peptidoglycan-binding proteins (Table S1) on contigs C3 and C9. These contigs also carried telomerase and terminal protein genes. Additionally, a frameshifted relaxase/mobilization nuclease domain-containing protein (RS38535/RS38540) and a MobC family relaxosome protein (RS33415) were encoded on C4. Both large circular plasmids (106,078 and 106,991 kb) contained relaxase genes (RS35115 and RS35870) and multiple conjugal transfer proteins (RS35590 and RS35825), suggesting that these plasmids were likely acquired via conjugation.

Beyond plasmid-encoded transfer systems, a MobF-type relaxase (RS00155) was identified within the “unknown origin” region of HGTi_I. A distinct set of conjugal transfer genes—including *tad*E (Flp pilus protein), type II secretion protein F, and *tad*A (conjugal transfer protein)—was located on chromosomal contig C2 (RS28260–RS28285). These genes are also conserved in other *R. erythropolis* genomes, indicating an ancestral chromosomal origin for part of the conjugal machinery.

Given the presence of “no database match” and plasmid-derived sequences, we systematically screened the NI86/21 genome for potential virulence factors using the virulence factor database (VFDB) and investigated antibiotic resistance determinants via the ResFinder web tool. These analyses yielded no hits, suggesting an absence of known virulence genes or acquired resistance markers. Additionally, phenotypic assessments showed that NI86/21 exhibits growth cessation above 32°C, a trait indicative of environmental rather than pathogenic adaptation (44).

Collectively, these results underscore that NI86/21 harbors a dynamic genome shaped by horizontal gene transfer but lacks identifiable virulence and resistance determinants, supporting its safety profile for biotechnological and environmental applications.

The mosaic-like organization of HGTi alongside both linear and circular plasmids within the NI86/21 genome is reflected in the variation of GC% content among different contigs and HGT regions, as detailed in Table S2. The average genomic GC% content of NI86/21 is 62.5%, consistent across the majority of contigs, with notable exceptions.

For example, HGTi_III displays a significantly reduced GC content of 58.6%, originating from the 0–80 kb “no match” region of the island (57.19 GC%) and the 80–148 kb prophage sequence (59.66 GC%) (Table S2). The elevated 64.7 GC% content of C3 can be attributed primarily to the incorporation of *M. frederiksbergense*-derived DNA sequences, which inherently possess a higher GC content of 65.7%. We also observed that those fragments within HGTi_V, C12, and C16, which were similar to *Nocardia*, *Gordonia*, *R. opacus,* or *R.* sp. chromosomal DNA stretches, had GC% content around 66%.

These findings collectively indicate that the NI86/21 strain has incorporated large exogenous DNA fragments exhibiting substantial GC% content divergence, reflecting complex recombination and HGT events. To assess the functional impact of such heterogeneity on gene expression, we investigated protein expression levels in these GC%-variable regions via proteomic analysis. Importantly, protein expression did not decline in regions with higher GC% content, such as the *M. frederiksbergense* DNA within contigs C3 and C4, where expression levels were 65% and 53%, respectively, while the average protein expression rate of the extrachromosomal genes was 53%.

The lower GC% content HGTi_III showed lower but sustained protein expression at approximately 36%. The 0–80 kb “no match” fragment had 43%, while the prophage region had 17% expression rate. Intriguingly, a subregion within the 0–80 kb region exhibiting 50 GC% (spanning genes RS15745 to RS15770) demonstrated complete (100%) protein expression coverage, underscoring the functional integration of even atypical genomic segments.

### Proteomic analysis of the NI86/21 strain and COG analysis of the core chromosome, HGTi, and extrachromosomal elements

Given the unusually large genome size of the NI86/21 strain, we monitored the expression profiles of the core chromosome, chromosomal HGTi, and extrachromosomal elements in rich medium under optimal growth conditions. LC-MS/MS analysis was performed using an Easy-nLC 1200 system coupled to a timsTOF Pro mass spectrometer. This approach enabled the identification of 4,817 proteins, representing approximately 67% of the 7,144 predicted protein-coding genes (Table 1). Protein expression rate of the core chromosome (72%) was significantly higher than the HGT element (51%). The expression rate of HGT islands and extrachromosomal elements was 47% and 53%, respectively, but when the prophage-encoded proteins and expressed phage proteins were subtracted, the HGTi expression rate was also 53%.

**TABLE 1:** Summary of gene and protein counts in *R. erythropolis* NI86/21, showing the number of predicted genes, LC-MS/MS-identified proteins, and proteomic coverage (%) across genomic compartments

	Genome	Core chromosome	HGT	Extrachromosomal	HGTi
Number of protein encoding genes (A)	7,144	5,652	1,492	941	551
Number of LC-MS/MS identified proteins (B)	4,817	4,050	758	499	259
Ratio of B/A in %	67%	72%	51%	53%	47%

**Fig 2:**
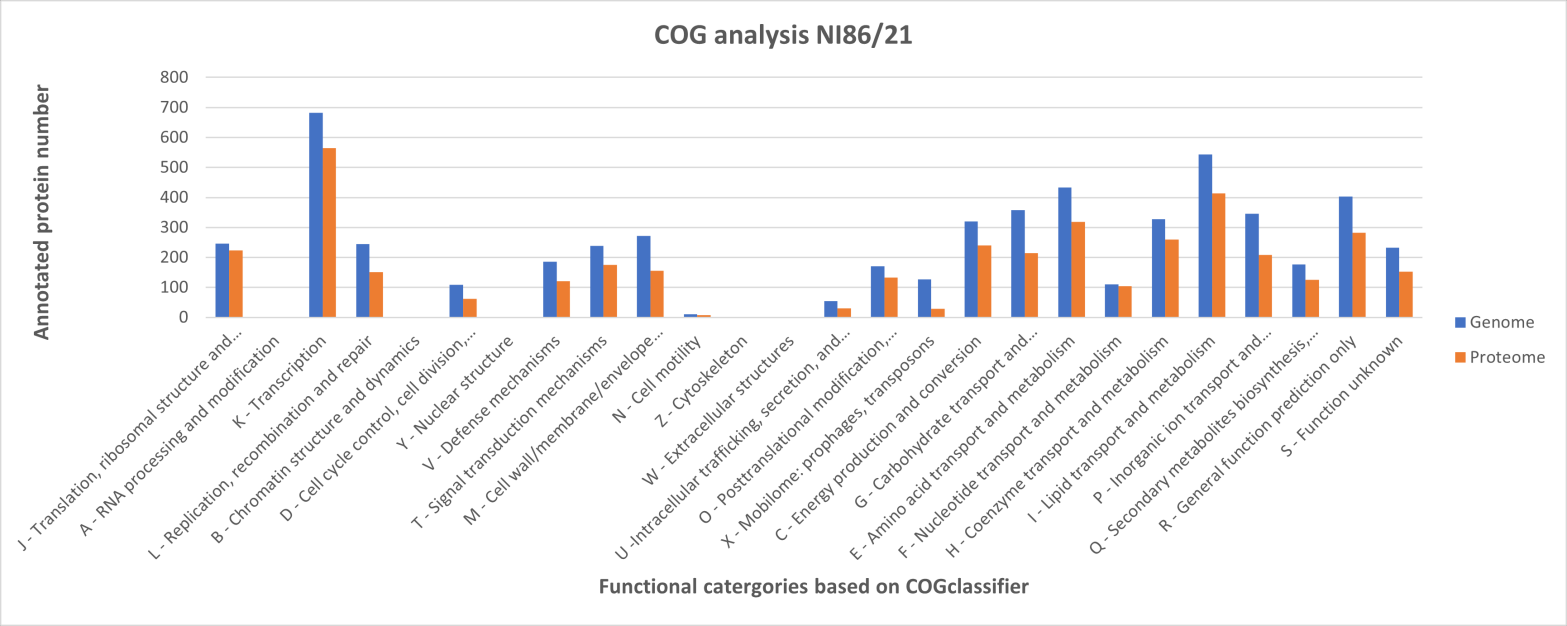
COG analysis of the annotated genome (blue bars) and the expressed proteome (orange bars) of the NI86/21 strain using the COGclassifier platform. Multiple functional groups show varying representation between genetic potential and actual protein expression.

Comprehensive COG annotation of the NI86/21 genome and proteome demonstrated a broad functional capacity (Fig. 2). In total, 5,596 proteins were involved in the COG analysis, of which 3,972 were detected by the proteome analysis. Transcription-related (K) proteins represented the largest functional class (682), followed by lipid transport and metabolism (544) (I), amino acid transport and metabolism (433) (E), and general function prediction (403) (R). Proteins involved in carbohydrate transport and metabolism (358) (G), energy production and conversion (320) (C), and inorganic ion transport and metabolism (345) (P) were also prominent. The largest discrepancies between the genome and proteome were observed in the categories related to mobilome: prophages and transposons (127 genomic vs. only 28 proteomic proteins); replication, recombination, and repair (245 vs. 151) (L); and cell wall biogenesis (272 vs. 156) (M), indicating that many proteins involved in genome plasticity and structural maintenance are not expressed under investigated conditions (Fig. 2). In contrast, core metabolic processes such as translation, ribosomal structure, and biogenesis (J), and nucleotide transport and metabolism (F) showed very small differences, reflecting their consistent activity to support growth.

**Fig 3:**
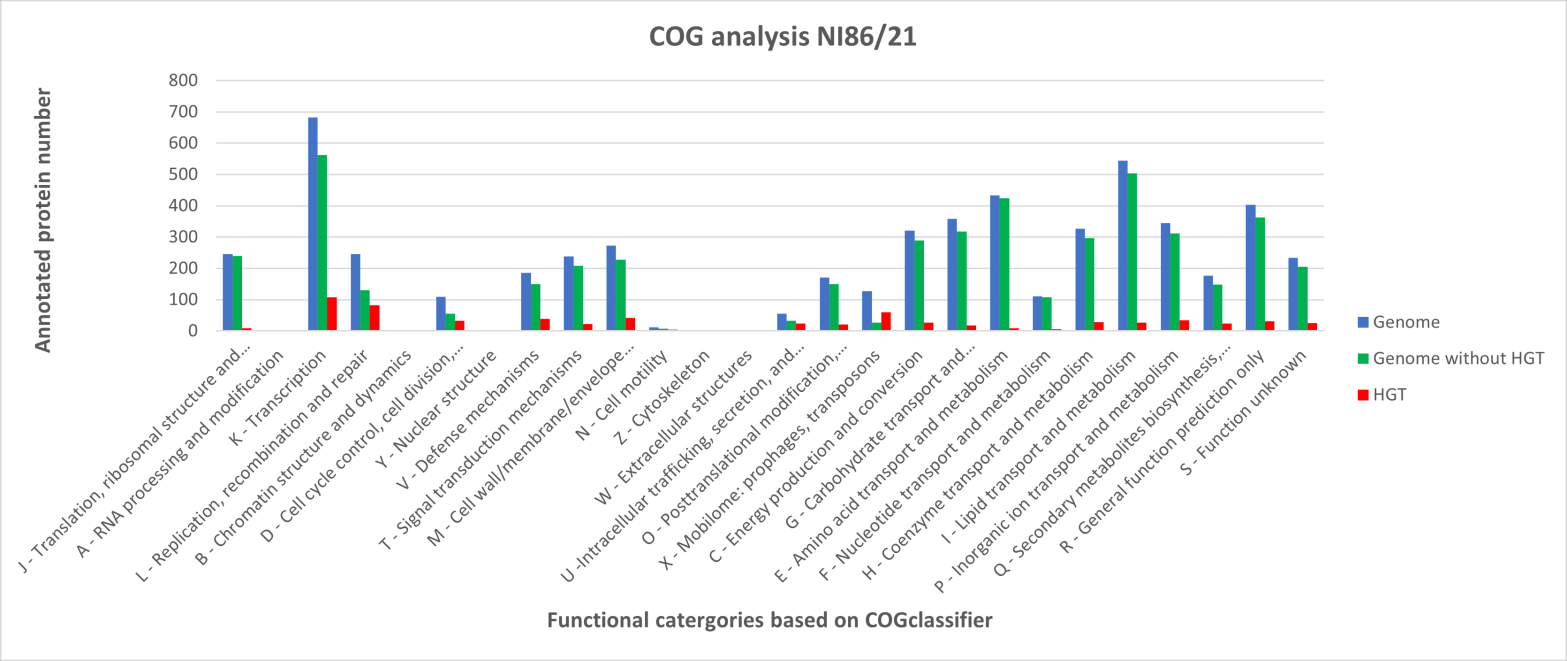
COG analysis of the genome (blue bars), the genome without HGTi and extrachromosomal elements (green bars), and the HGTi and extrachromosomal elements together (red bars) of the NI86/21 strain.

Comparison between the genome without HGT proteins and the HGT-associated sequences revealed distinct functional enrichment patterns (Fig. 3). Only 668 (45%) out of the 1,472 proteins encoded on HGT elements could be sorted to COG categories. In contrast, 83% (4,754 of 5,733) of the ORFs in the core chromosome were classified into functional categories. This marked difference was mainly due to the high proportion of hypothetical proteins within the HGT regions (approximately 45%), compared to only 10% in the CC.

HGT proteins in COG categories L; D; intracellular trafficking, secretion, and vesicular transport (U); and X provided the highest HGT/core chromosomal protein ratio differences, with the notion that the HGT’s mobilome portion exceeded the chromosomal portion. The increase of HGT proteins in categories L, D, U, and X is attributed to genes of phage and plasmid maintenance, transfection, and conjugation and the jumping genetic elements. HGT proteins in translation, ribosomal structure, and biogenesis (J); nucleotide transport and metabolism (F); and amino acid transport and metabolism (E) categories were negligible.

### Recombinases, integrases and transposases of the NI86/21 strain

Jumping genetic elements of NI86/21 are distributed across the chromosome, plasmids, and HGTi, totaling 140 recombinases, integrases, and transposases, of which 67 exhibited protein expression. Recombinases show the highest expression rates overall, with 80% active across all regions, followed by integrases at 50%. Transposases, while the most abundant (91), display moderate expression levels at 43%. Other elements, such as insertion/recombination (I/R) and insertion/recombination/transposition (I/R/T) factors, are less prevalent and show low or no expression. Overall, approximately 48% of these proteins are expressed in the NI86/21 strain, and jumping elements themselves were not analyzed (except the novel Tn7706 transposon), but the expression of enzymes that facilitate their movement was examined (Table 2 ;Table S3).

**TABLE 2:** Distribution and expression of transposases (T), integrases (I), and recombinases (R) across the chromosomal, plasmid, and HGTi regions of the NI86/21 genome^*a*^

	Chromosome			Plasmid			HGTi			Total		
	TP	EX	%	TP	EX	%	TP	EX	%	TP	EX	%
Recombinase	5	5	100	6	5	83	9	6	67	20	16	80
Integrase	3	2	67	10	6	60	7	4	57	20	10	50
Transposase	17	10	59	28	11	39	46	18	39	91	39	43
I/R	2	1	50	4	1	25	1	0	0	7	2	29
I/R/T	0	0	0	1	0	0	1	0	0	2	0	0
Total	27	18	67	49	23	47	64	28	44	140	67	48

^*a*^ The table shows the total number of proteins (TP), the number expressed (EX), and the percentage (%) of expressed proteins within each genomic category.

**Fig 4:**
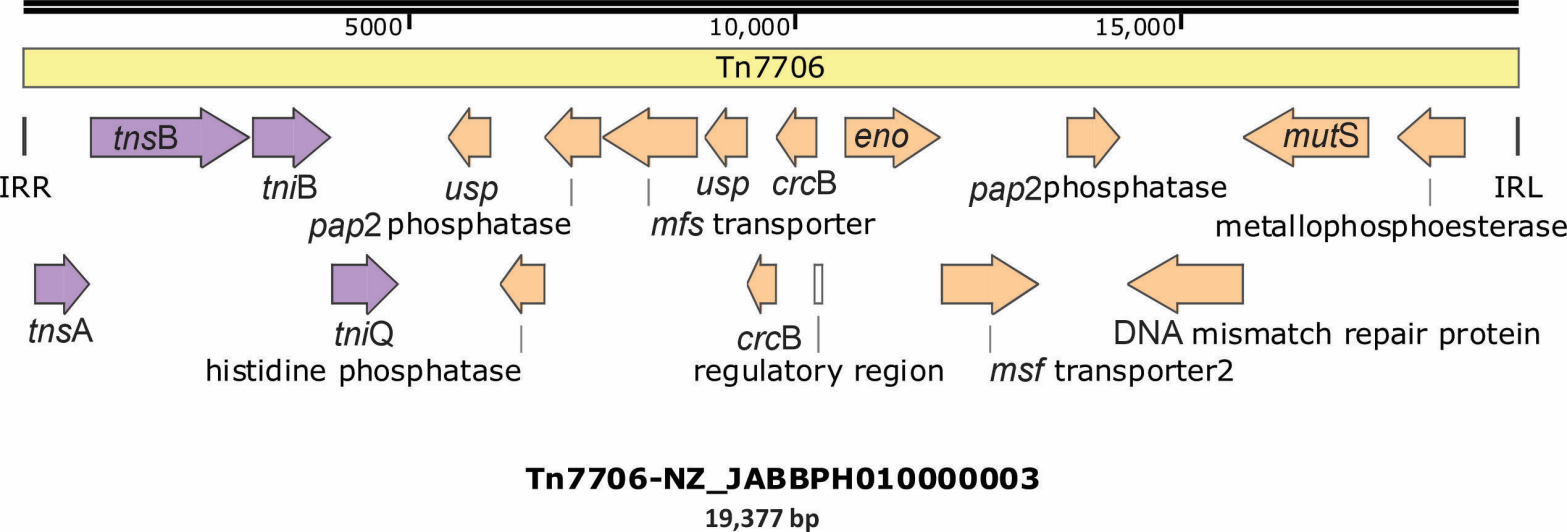
Gene organization of the Tn7706 transposon. Inverted repeats at the transposon ends are shown as black rods, open reading frames (ORFs) involved in transposition are highlighted in violet, and passanger ORFs are indicated in orange.

**Fig 5:**
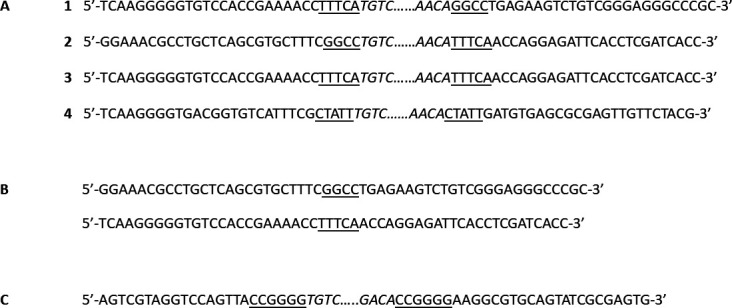
Integration sites of complete Tn7706 copies in contigs C12, C11, C13, and C3 (**A**). Reconstruction of original sequences by exchanging right-end regions between C12 and C11 (**B**). IS1416 insertion within Tn7706 on plasmid pYYL2 (**C**). Inverted repeats are italicized; direct repeats are underlined.

The most prominent transposable element was the multicopy transposon Tn7706, measuring 19,377 bp. Partial and/or complete copies were detected in the majority of contigs, either at the 5′ or 3′ end of the contig or embedded internally (Table 3). To facilitate broader access and annotation, Tn7706 sequences were also submitted to the TnCentral (https://tncentral.ncc.unesp.br/report/te/Tn7706-NZ_JABBPH010000003). Tn7706 encodes 19 genes, including the transposition-related genes *tns*A, *tns*B, *tni*Q, and *tni*B, which are characteristic of Tn7-like transposons (Fig. 4). Integration sites of full-length Tn7706 copies are indicated in Fig. 5.

**TABLE 3:** Occurrence and location of the Tn7706 transposon in nine contigs of NI86/21 genome^*a*^

Contig	C1	C2	C3	C4	C5	C9	C11	C12	C13
Position	3′	5′, 3′	I	5′	3′	5′	I	I	I

^*a*^ Only internally located copies (I) represent full-length transposons. Partial copies are found at the 5′ and at the 3′ ends of contigs.

When the left and right end regions of Tn7706 encoded on contigs C11 and C12 were exchanged, they showed 100% sequence identity to plasmid pCP61 from *R. qingshengii* strain A29-k1 and plasmid pRLLBG43 from *R. erythropolis* strain BG43, respectively. This finding suggests that extrachromosomal elements may undergo recombination through cross-over events mediated by Tn7706 DNA fragments. BLASTN analysis identified the presence of the Tn7706 transposon integrated into plasmids of five closely related NI86/21 isolates, specifically *R. erythropolis* X5 (plasmid pRhX5), *R. qingshengii* JCM 15477 (plasmid pdjl-6-5), *R. qingshengii* CX-1 (plasmid unnamed2), *Rhodococcus* sp. DN22 (plasmid p1), *Rhodococcus* sp. YL-1 (plasmid pYLL2), and *R. qingshengii* F2-2 (plasmid pLP337, partial). Notably, the Tn7706 copy integrated into the pYLL2 plasmid was extended by 2,577 bp due to the insertion of an IS21-family element, IS1415, at position 5,824, along with a 6 bp direct repeat (CCGGGG), resulting in a total length of 21,960 bp for this transposon copy.

### HGTi_III encodes a prophage sequence

The phage detection tool Phastest on the Proksee platform identified one intact prophage with 66.789 bp size, constituting the right end of HGTi_III (39–41). The presence of attachment site sequences (*tttttcgtgccc*), the vicinity of a tRNA gene (RS16505), and genes like type IV secretory system conjugative DNA transfer family protein, replication-relaxation family protein, antirestriction protein ArdA, phage tail tape measure protein, phage tail tube protein, minor capsid protein, phage minor capsid protein, RusA family crossover junction endodeoxyribonuclease, phage recombination protein Bet, YqaJ viral recombinase family protein for phage assembly and infection encoded in the region from RS16045 to RS16500 (Table S1 - Phage-related seq) are evidence for a *Rhodococcus* prophage sequence. Since Mitomycin C has been validated for the induction of prophages in *Mycolata* (45), we evaluate our observation that NI86/21 cells lyse when mitomycin C is added to stationary phase cells (44) as evidence for the lytic life cycle of this temperate phage. Interestingly, this phage bears one out of the six copies of the IS1415 insertion element. Whether the phage imported the IS1415 into the NI86/21 cells or the IS1415 jumped into the phage after infection remains elusive.

The proteomic analysis detected the expression of only 15 out of 85 (17%) putative phage proteins (Table S1 - Phage-related seq).

### Toxin-antitoxin systems of the NI86/21 strain

Genome annotation of the NI86/21 strain revealed a diverse and abundant repertoire of toxin–antitoxin (TA) systems (Table S1). In total, 28 TA-related genes were identified, comprising 15 toxin genes and 13 antitoxin genes distributed across both type II and type IV systems. Among type II systems, the PemK/MazF family was the most prevalent, with five toxin-encoding genes detected. Additional type II toxins included members of the RelE/ParE (two occurrences) and VapC (two occurrences) families, along with single representatives of the Phd/YefM antitoxin and zeta toxin families. Type II antitoxins also included VapB and HicB family proteins. Type IV systems were also prominent, comprising five AbiEi family antitoxin domain-containing proteins and three nucleotidyl transferase AbiEii/AbiGii toxin family proteins. Furthermore, a single VbhA family antitoxin was identified. Several genes were annotated as general toxins or antitoxins without specific family assignments. In total, TA systems represented the PemK/MazF, RelE/ParE, VapB, VapC, HicB, Phd/YefM, AbiEi/AbiGii, VbhA, CapL, and zeta toxin families. We analyzed the genomic distribution of these loci and found that three antitoxins (two HicB antitoxins and one VapB antitoxin) and a PemK/MazF toxin–antitoxin pair were encoded in genomic–prophage (G–PP) regions, while the remaining genes were located in plasmid-associated (PP) regions. Proteomic analysis demonstrated that 27 out of the 28 TA-related genes were expressed under the conditions tested, underscoring their likely active role in maintaining genome stability, plasmid persistence, and adaptation to environmental stress.

### Defense systems of the NI86/21 strain

In the genome of the NI86/21 strain, we identified a putative prophage using the Phastest platform, suggesting prior phage exposure and integration events. To further explore the strain’s capacity to counteract phage attacks, we screened the genome for phage defense systems using DefenseFinder (46). Remarkably, this analysis revealed a total of 21 distinct defense systems dispersed throughout the genome, highlighting a robust and multifaceted phage resistance repertoire (Table 4). Among these, three CRISPR-Cas Class 1, Subtype IV-B systems were detected, encoding RAMP superfamily CRISPR-associated proteins (RS32496, RS36625, and RS37095). Notably, these proteins are localized on linear plasmid contigs (C3, C8, and C10), a feature conserved among related *R. erythropolis* and *R. quingsengi* strains as indicated by similarity searches.

**TABLE 4:** List of identified phage defense systems in the NI86/21 genome^*a*^

Defense system type	Defense system subtype	Related protein ID
Ceres	Ceres	MBY6382328.1
Gabija	Gabija	MBY6382918.1, MBY6382919.1
RM	RM_Type_IIG	MBY6382921.1
Wadjet	Wadjet_II	MBY6384608.1, MBY6384609.1, MBY6384610.1
Gabija	Gabija	MBY6385362.1, MBY6385363.1
RM	RM_Type_II	MBY6385366.1, MBY6385368.1
SpbK	SpbK	MBY6385389.1
Ceres	Ceres	MBY6386573.1
Eleos	Eleos	MBY6387038.1, MBY6387039.1
RM	RM_Type_I	MBY6387324.1, MBY6387325.1
AbiAlpha	AbiAlpha	MBY6388060.1
Ceres	Ceres	MBY6388195.1
PD-T4-3	PD-T4-3	MBY6388375.1
RM	RM_Type_IIG	MBY6388521.1
AbiE	AbiE	MBY6388531.1, MBY6388532.1
Cas	CAS_Class1-Subtype-IV-B	MBY6388620.1, MBY6388621.1, MBY6388622.1, MBY6388623.1, MBY6388624.1
AbiE	AbiE	MBY6388688.1, MBY6388689.1
Ceres	Ceres	MBY6389337.1
Ceres	Ceres	MBY6389366.1
Cas	CAS_Class1-Subtype-IV-B	MBY6389415.1, MBY6389416.1, MBY6389417.1, MBY6389420.1
Cas	CAS_Class1-Subtype-IV-B	MBY6389507.1, MBY6389508.1, MBY6389510.1, MBY6389511.1

^*a*^ For each defense system type and subtype, associated protein accession identifiers are provided, illustrating the genetic basis of the strain’s antiviral defense arsenal.

### Proteins putatively involved in bioconversion and degradation processes

The NI86/21 strain exhibits extensive bioconversion and degradation capabilities, supported by a remarkably rich enzymatic repertoire encoded within its genome. Genomic analysis identified over 1,000 genes putatively involved in key catabolic processes, including oxidations, dehydrogenations, epoxidations, hydrolysis, hydroxylation, dehalogenations, and desulfurization (Table 5). These enzymes predominantly belong to major functional classes such as oxidoreductases, hydrolases, dehydrogenases, reductases, and various oxidases—including peroxidases, dioxygenases, and monooxygenases—underscoring the strain’s diverse metabolic potential.

**TABLE 5:** Genomic and proteomic counts and expression percentages of enzymes involved in bioconversion and degradation in *R. erythropolis* NI86/21^*a*^

Protein family	Genome			Chromosome			Plasmid			HGTi			Percentage distribution (%)
	ORF	Exp.	%	ORF	Exp.	%	ORF	Exp.	%	ORF	Exp.	%	
Oxidoreductase	289	199	69	271	187	69	8	5	63	10	7	70	26.73
Hydrolase	257	204	80	233	185	79	19	14	74	5	5	100	23.77
Dehydrogenase	235	194	83	220	182	83	8	6	75	7	6	86	21.74
Reductase	105	93	89	90	79	88	12	8	66	3	2	66	9.71
Oxygenases	84	51	61	77	44	57	2	2	100	5	5	100	7.77
Oxidase	58	44	76	48	39	81	9	4	44	1	1	100	5.37
Cytochrome P450	22	11	50	10	4	40	8	5	63	4	2	50	2.04
Catalase	4	4	100	3	3	100	0	0	0	1	1	100	0.37
Hydroxylase	4	3	75	3	2	67	0	0	0	1	1	100	0.37
Dehalogenase	3	3	100	3	3	100	0	0	0	0	0	0	0.28
Resistance	20	13	65	12	9	75	7	4	57	1	0	0	1.85
Total	1,081	819	76	970	737	76	73	48	66	38	30	79	100
Hypothetical	1,264	547	43	584	278	48	460	205	45	220	64	29	0

^*a*^ Expression status is denoted as Exp. if expressed. Functional categories are grouped as follows: oxidases include oxidases, peroxidases, and epoxidases; oxygenases include oxygenases, monooxygenases, and dioxygenases.

Proteomic profiling under standard growth conditions confirmed the expression of approximately 75%–78% of these predicted proteins, indicating active engagement of these pathways. For example, among oxidoreductases, roughly 72%–69% of genes were expressed, and hydrolases showed even higher expression rates (81%). Dehydrogenases exhibited the greatest coverage, with over 85% detected proteomically, highlighting their central role in metabolic flux. Additionally, cytochrome P450 enzymes displayed moderate expression (53%), while dehalogenases were fully expressed, emphasizing the strain’s capability in xenobiotic transformation and environmental detoxification.

The genomic distribution of these enzymes is predominantly chromosomal (~90%), with a smaller yet significant contribution from plasmids and HGTi (Table S4).

### Hypothetical proteins

We analyzed the genomic distribution of the 1,264 hypothetical proteins and found that 584 (46%) are located on the chromosome, while the remaining 680 (54%) are associated with plasmids (460 proteins) and HGTi (220 proteins). These hypothetical proteins represent 11%, 49%, and 40% of the coding sequences within the chromosome, plasmids, and HGTi, respectively, suggesting a gradual assimilation process. Proteomic data revealed relatively low expression rates for these proteins—48% on the chromosome, 44% on plasmids, and 30% on HGTi.

### Secondary metabolites

To demonstrate that HGT events might affect secondary metabolite productions, we describe here the richness of the genome in non-ribosomal peptide synthase and polyketide synthase genes. Non-ribosomal peptides and polyketides are a diverse group of natural products with complex chemical structures and enormous pharmaceutical potential. They are synthesized on modular NRPS and PKS enzyme complexes by a conserved thio template mechanism (47).

**Fig 6:**
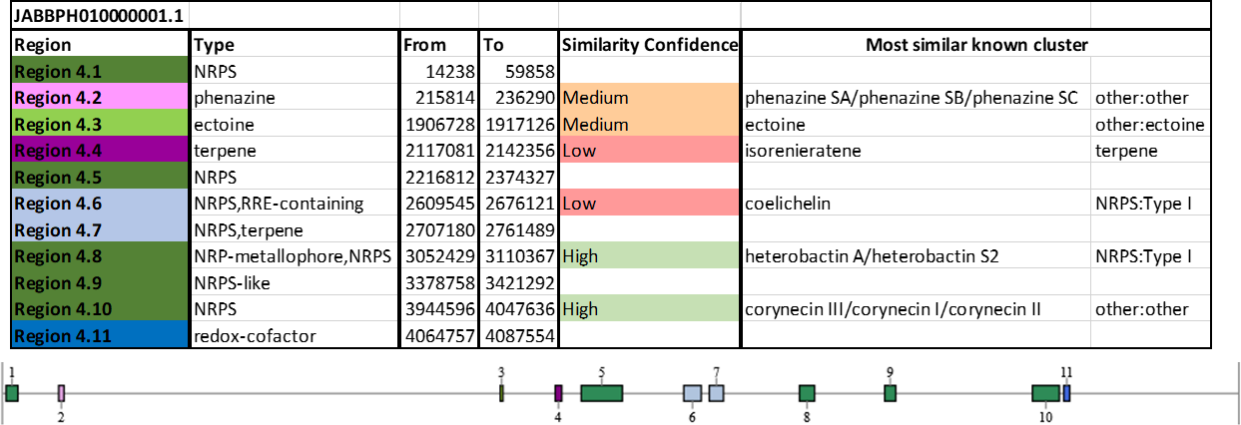
Distribution and classification of secondary metabolite biosynthetic gene clusters identified in *R. erythropolis* NI86/21 chromosomal Contig 1 by anti-SMASH.

**Fig 7:**
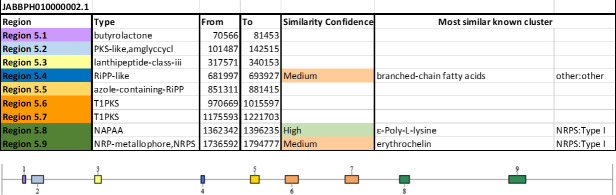
Distribution and classification of secondary metabolite biosynthetic gene clusters identified in *R. erythropolis* NI86/21 chromosomal Contig 2 by anti-SMASH.

Genome mining with the anti-SMASH webserver identified 20 secondary metabolite biosynthetic gene clusters in NI86/21, all located on chromosomal Conig1 (Fig. 6) and on chromosomal Contig2 (Fig. 7) . Among these, three clusters were associated with horizontally transferred genomic regions. Notably, Contig 1-Region 4.1 (14,238–59,858 bp) showed no similarity to any known secondary metabolite, suggesting a potentially novel pathway, although its core biosynthetic genes shared 75%–86% amino acid identity to polyketide synthases and NRPSs from *Williamsia soli* and *R. fascians*. Contig 1-Region 4.2 (215,814–236,290 bp) encoded a phenazine biosynthetic gene cluster (56% similarity) similar to plasmid sequences of *R. erythropolis*. The third HGT-associated region (cluster 5.8) contained large NRPS/PKS genes nearly identical (94%–100% identity) to ε-poly-L-lysine producers.

Other clusters included loci with high similarity to heterobactin (siderophore) and corynecin (polyketide) pathways, medium similarity to ectoine, erythrochelin, and branched-chain fatty acid biosynthesis, and several clusters without clear similarities, highlighting a rich and partly unique secondary metabolite repertoire.

Proteomic analysis confirmed that nearly all biosynthetic genes were expressed, indicating active production potential. This supports the antimicrobial and metabolic versatility of the strain, driven by diverse polyketide and nonribosomal peptide pathways (48).

### Subunit orthologs of the ribosome

Having the example of the 20S proteasome, where a truncated operon of proteasomal genes was acquired by HGT, we monitored whether the NI86/21 strain could acquire ribosomal subunit genes by HGT. We filtered the unique gene set (7,144 proteins) for ribosomal subunits and identified eight (S14, S18, L11, L28, L31, L32, L33, and L7/L12) present in two or more copies. One set of S14, S18, L11, L28, L31, L32, and L33 is encoded in an operon-like cluster (RS05945–RS05975). Additional copies were located elsewhere: S18 (RS24665 and RS18205), L28 (RS15250), L31 (RS08385), L32 (RS15210), L33 (RS19180), and L11 (RS19150). The L7/L12 subunit (RS19130) was also found as a single ORF outside the operon. Notably, RS38780 (encoding the 50S ribosomal protein L11 methyltransferase) is the only ribosomal gene located within an HGTi (HGTi_I). Comparison to the *R. erythropolis* PR4 and R85 genomes revealed similar duplications, except that L7/L12 and L11 are not duplicated there. Expression analyses showed that, except for RS05945, the operon genes are not expressed under the tested conditions, while the additional copies of S18, L7/L12, and L11 are expressed. These data indicate that similarly to the non-essential 20S proteasome, the ribosome of NI86/21 exhibits heteromorph quaternary structures due to duplication and differential expression of ribosomal protein genes likely shaped by horizontal gene transfer (Table S6).

### Cytochrome P450-dependent EPTC and atrazine degradation system is encoded on a horizontally acquired island and shares a near-identical sequence with a Canadian isolate

The inducible cytochrome P450 monooxygenase (CYP116 family, previously designated ThcB/EptA) that catalyzes the initial oxidation and N-dealkylation of the thiocarbamate herbicide EPTC and N-dealkylation of atrazine in *R. erythropolis* NI86/21 (24) is encoded within the 115 kb horizontally acquired island HGTi_V (coordinates 55–82 kb, Table S2). The cluster includes the structural gene *thc*B, the LysR-type regulator *thc*R, and downstream genes including a deamidase/depupylase (*dop*) and a truncated proteasome operon (*pgs*A–*paf*A–*pup*). Although the complete sequence of the ~77 kb conjugative plasmid responsible for EPTC and atrazine degradation in *Rhodococcus* sp. TE1 (isolated in Canada in the 1980s) has never been published, two diagnostic regions are publicly available and were directly compared; the comparison of two diagnostic regions revealed near-identical sequences with NI86/21. The GenBank entry U32870.1 (1,153 bp), covering the 3′ end of *thc*R and the flanking *dop* gene, is 100% identical to the corresponding region in NI86/21. Sanger sequencing of the *thc*B gene from TE1 showed only a single nucleotide difference relative to NI86/21 (R. Behki, personal communication). The same co-linear arrangement of the truncated proteasome operon upstream of *thc*R–*dop*–*thc*B is observed in TE1. 2D PAGE-based proteomic analysis confirmed the inducible expression of ThcB and downstream proteins when the cells were exposed to EPTC (49). The identical herbicide-degrading ability and near-perfect sequence similarity between TE1 and NI86/21 demonstrate independent acquisition of the same HGT-encoded catabolic module under identical selective pressure. This finding highlights the role of horizontally acquired genes in conferring adaptive metabolic capabilities and the evolutionary predictability of herbicide-degrading traits in *Rhodococcus* (Table 6).

**TABLE 6:** Parallel phenotypic and genetic features of the EPTC/atrazine degradation system in *Rhodococcus* sp. TE1 (Canada) and *R. erythropolis* NI86/21 (Hungary)

Aspect	*Rhodococcus* sp. TE1 (Canadian isolate)	*R. erythropolis* NI86/21 (Hungarian Isolate)
Isolation source	Thiocarbamate-treated agricultural soil (Ontario, Canada; in the 1980s)	Maize rhizosphere soil exposed to thiocarbamate herbicides (Hungary, 1986)
Primary phenotype	Degrades EPTC and uses it as sole carbon source	Degrades EPTC and uses it as sole carbon source
Secondary phenotype	Degrades atrazine and uses it as sole carbon source; co-degrades EPTC + atrazine efficiently	Degrades atrazine and uses it as sole carbon source. Co-degrades EPTC + atrazine efficiently
Genetic basis	An indigenous ~77-kb conjugative plasmid encodes for the *ept*A/*atr*A (EPTC and atrazine degradation/N-dealkylation enzyme) on a 6.2-kb *Kpn*I fragment; no expression in *E. coli*; transferable to other *Rhodococcus* strains via shuttle vector (pBS305); the module is associated with 20S proteasome accessory genes	The chromosomal HGT_V island (~115 kb) encodes for the catabolic EPTC and atrazine degrading module (*thc*B/*ept*A/*atr*A, *thc*C, *thc*D and *thc*R); the module is associated with the truncated proteasome *pgs*A–*paf*A–*pup* operon; ThcB is up-regulated upon herbicide exposure
Key enzymes/genes	*ept*A/*atr*A: the same P450 system (*thc*B*, thc*C, *thc*D and *thc*R) is mentioned, as in case of NI86/21; the module is associated with proteasome accessory genes (partial sequencing)	CYP116/ThcB: inducible P450 for EPTC oxidation and N-dealkylation, and atrazine dealkylation. *thc*R: LysR-type regulator. *dop* + truncated proteasome operon (*pgs*A–*paf*A–*pup*) in the flanking region of *thc*R; 100% identical to TE1 fragment (U32870.1); single nucleotide difference in *thc*B (50)
Ecological/adaptive role	Enables survival in herbicide-contaminated agro-soils; plasmid mobility suggests HGT hotspot in *Nocardiaceae*	Rhizosphere specialist; HGT-driven plasticity (8.046 Mb genome with 0.64 Mb HGT islands) confers metabolic versatility; active in mixed herbicide degradation, partial silencing of foreign genes for stability
Evidence of parallelism	Identical nucleotide sequences in *thc*B and *thc*R-*dop* fragments, >7,000 km apart; same selective pressure (thiocarbamate/atrazine) drives convergent HGT acquisition	

## DISCUSSION

Soils are highly dynamic ecosystems in which biotic and abiotic conditions can shift rapidly, and soil microbes must have the ability to survive extreme temperatures, low water capacity, and limited mineral supplies, and effectively outcompete other organisms for limited nutrients. Most soil microbes produce diverse secondary metabolites such as antibiotics, antifungals, quorum-sensing molecules, and siderophores that mediate communication, competition, and interactions with other organisms and the environment. *R. erythropolis* exemplifies this ecological versatility: *R. erythropolis* strains are typical mesophilic soil bacteria that can grow at very low temperatures (51), can survive drought, can produce NRPs and steroids (52), have antifungal properties (53), produce siderophores, and importantly can degrade secondary metabolites like quorum-sensing molecules (54), mycotoxins (30), and many other chemicals present in the environment in a broadly permissive, “take-all-comers” manner. *R. erythropolis* strains encode approximately a thousand enzymes potentially involved in bioconversion and biodegradation processes. In addition to chromosomally encoded genes, strain NI86/21 has acquired an extensive repertoire of genes through horizontal gene transfer, comprising 1,497 genes located on 1.77 Mb of horizontally acquired DNA. Acquiring this amount of genetic material has advantages and disadvantages for the cell. Plasmids encoding enzymes can provide opportunities to outcompete microbial competitors, but they also can increase cell division time, and bringing in excess of transposable elements can lead to genome rearrangements and cell death (55, 56). Therefore, bacterial cells have to balance between gaining and losing HGT elements (57) to be fit and competitive. The NI86/21 genome reflects this tension clearly: the mosaic-like structure of the HGT islands and the plasmids, the high number of expressed transposases, integrases, and recombinases indicate that the genome of *R. erythropolis* NI86/21 is under pressure of continuous recombination events. Proteomic data showed that ~50% of integrases and transposases were expressed under the examined conditions, potentially causing high mutation rates. This inference is supported by the presence of nine copies of the newly identified Tn7706 transposon (three encoded on HGTi and six on plasmids), while in other *R. erythropolis* strains, the Tn7706 transposon is found only on linear plasmids. Another example supporting active transposition for the expanded copy number of an IS element, IS1416, which duplicated its copy number during lab passages, since we detected three copies in 1997 (29), and six copies in the genome sequence.

We found five HGTi in the large chromosomal contig of the NI86/21 strain. HGTi are not exclusive to NI86/21; other *R. erythropolis* strains also harbor such regions. The chromosome of the PR4 strain contains five HGT islands (Fig. 1). The ~91-kb HGTi_I, spanning proteins RS05825–RS06230, has no DNA-level matches, but protein-level analysis shows similarity to nocardioform bacteria. This region is enriched in oxidoreductases, reductases, CYP450s, and other bioconversion-related proteins (34/83), with relatively few hypothetical proteins (7/83) and no insertion elements. HGTi_II and HGTi_III encode an intact prophage (2.44–2.49 Mb) and an incomplete prophage (2.695–2.706 Mb), respectively. The 5′ region of HGTi_IV contains mostly hypothetical “no-match” proteins (28/36), while the 3′ region encodes the Brx1 antiphage system (BrxA, BrxB, BrxC, PglX, PglZ-A, and BrxL) with low identity to actinomycetal homologs. HGTi_V (3.705-3.757 Mb) shows similarity to both chromosomal (*R. erythropolis* XP, *R.* sp. DN22, *R. qingshengii* CX-1) and plasmid sequences (*R.* sp. BH4, *R. qingshengii* TG-1, unnamed plasmid1). Another example of an HGT island in *R. erythropolis* is the 45–88 kb chromosomal region of the R85 strain, which lacks DNA-level similarity but shows low amino-acid identity to actinomycetal ORFs. Together, these observations support that plasmid- and chromosome-derived actinomycete DNA fragments can integrate into recipient *R. erythropolis* genomes, contributing to their genomic diversity.

The genome stability of strain NI86/21 is supported by 29 toxin-antitoxin proteins (28 expressed) that maintain plasmids and the genome. Six restriction-modification systems were identified, with four expressed. Notably, one enzyme pair (RS35255 and RS35260) likely acquired by HGT is encoded on a large circular plasmid (Contig 6). Collectively, the abundance and diversity of TA systems in NI86/21 underscore the complex regulatory networks this strain deploys to ensure survival and genomic integrity. Despite the presence of 21 defense systems (including three Cas systems), we found evidence for the presence of an intact prophage. Since the CRISPR homologs were encoded on linear plasmid contigs, it is plausible that the phages invaded the NI86/21 cells before the arrival of the linear plasmids.

The horizontally acquired regions of the genome displayed mosaic-like structure, incorporating DNA primarily from *Rhodococcus* species and also containing substantial segments derived from *Actinomycetes* and uncharacterized sources. Protein-level searches of these “no match” regions identified only a limited number of hits, typically exhibiting low amino acid identity, mostly with nocardioform bacteria. Notably, a few proteins—such as RS15755 and RS15760—showed similarity to sequences from *Cyanobacteria*, *Mesorhizobium*, *Pseudomonas*, *Thiobacillus*, and *Amycolatopsis* species.

The 62.5% GC content of the NI86/21 genome was generally consistent throughout the contigs, with the exception of C3 (65%) and HGTi_III (58.6%). These findings suggest that the strain is capable of acquiring and maintaining DNA fragments with markedly divergent GC% composition. To assess whether such regions are transcriptionally active, we examined the expression of genes within a segment of HGTi_III characterized by particularly low GC content (~50%). Proteomic analysis confirmed that genes from this region are indeed expressed, showing functional integration of the foreign DNA. This pattern supports the hypothesis that newly acquired genes undergo a functional “trial period” during which they adapt to the host genome’s regulatory and compositional context, eventually increasing their stability, modulating their expression, and potentially gaining or altering functionality (58).

Due to the high number of hypothetical proteins, only 676 of the 1,497 ORFs of the HGT DNA could be assigned to COG categories. Apparently, genes for replication and translation were rarely transferred, although an L11 ribosomal subunit sequence (RS38780) was found on HGTi_I.

Plasmid- and HGT-encoded biodegradation-related proteins were less frequent than those on the core chromosome (7% vs. 17%). Instead, the HGT DNA portion of NI86/21 harbors a high number of hypothetical proteins.

We have found evidence that expression of HGT genes could be constitutive, as it was demonstrated on the example of the NI86/21 20S proteasome (18), leading to an intermediate complexity positioning this particle between the eukaryotic 26S core particle and prokaryotic 20S proteasomes, and heterologous population (59). Heterogenic macromolecular populations are not limited to the 20S proteasome in the NI86/21 strain, since we found evidence that two L7/L12 and three S18 ribosomal subunit homologs are expressed, leading to heteromorph quaternary structures of the ribosome. This observation is not exclusive to *R. erythropolis* strains, as Teramoto and coworkers also reported heterogeneous quaternary ribosome structures based on a matrix assisted laser desorption/ionization time of flight mass spectrometry (MALDI-TOF-MS) analysis of ribosomal subunit composition (60).

Inducible proteins in two HGTi of the NI86/21 chromosome were found since the EPTC thiocarbamate could induce the expression of the CYP450 (ThcB, RS21800, encoded on HGTi_V) and a chloroperoxidase enzyme (ThcF, RS00470, encoded on HGTi_I). The arrival of the CYP450 gene/protein (catalyzing the first, dealkylating step of the herbicide) opened the gate for a new xenobiotic degradation pathway. Downstream of the pathway, the aldehyde dehydrogenase (ThcA, RS18690) and the NDMA-dependent alcohol oxidoreductase (ThcE, RS18850) acted on the cleaved-off alkyl groups, and they were encoded on the core chromosomal part of the genome. The regulation of ThcB and ThcE is different from that of ThcA and ThcF enzymes. Ethanol amine could induce only ThcA, atrazine herbicide induced ThcA, ThcB, and ThcE, while EPTC induced all of them (49). The role of the ThcF enzyme remains elusive, since the expression of the NI86/21 counterpart (RS09885) was not affected by thiocarbamates. However, there are indications that ThcF-like enzymes in *Streptomyces* play a role in maintaining the healthy homeostasis of the cell (61).

The DNA fragment encoding the *thc* gene cluster and the truncated proteasome operon of NI86/21 showed the highest similarity to corresponding genes of *Rhodococcus* sp. USK10, which is encoded on its 8.4-Mb, high-GC chromosome (67%). Unlike the USK10 arrangement, the NI86/21 *thc* cluster is inverted and lacks a ~12-kb segment originally located between the *thc* genes and the proteasome operon, indicating structural rearrangement after acquisition. Shao and Behki described the isolation and expression of EPTC- and atrazine-degrading genes from *Rhodococcus* sp. TE1 (isolated from EPTC-treated soil in Canada), which they subcloned from a 77-kb plasmid (62). Unfortunately, the DNA sequence was not published, but the DNA fragment encoding for the *thc*B gene was identical to the NI86/21 counterpart except one base (R. Behki personal communication). Moreover, the authors deposited a 1,153-bp fragment (GenBank: U32870.1) corresponding to the “*thc*R gene, 3′' flanking sequence,” which is identical to the depupylase/deamidase (*dop*) gene of the NI86/21 truncated proteasome operon. This strongly suggests that the EPTC-degrading *thc* cluster and the associated proteasome genes in both NI86/21 and TE1 were acquired from the chromosome of a high-GC% content *Rhodococcus* species via HGT. The identical nucleotide sequences of the *thc*B monooxygenase gene and the *thc*R-*dop* fragment in *R. erythropolis* NI86/21 and *Rhodococcus* sp. TE1—strains isolated more than 7,000 km apart—provide compelling evidence that the same ~25–30-kb catabolic module has been acquired independently in two distant *Rhodococcus* populations subjected to identical thiocarbamate herbicide selection (Table 2). In TE1, the module resides on a conjugative plasmid, whereas in NI86/21, it is chromosomally integrated within a larger mosaic-like HGTi, illustrating two alternative stable endpoints of the same mobile element. The co-transfer of proteasome accessory genes (*pgs*A–*paf*A–*pup*) with the catabolic operon in both strains further supports the existence of a single, composite mobile element that couples herbicide degradation with protein quality-control functions. This case represents one of the most striking documented examples of parallel evolution of a complex, multigene catabolic trait in environmental bacteria driven by anthropogenic chemical pressure.

The incorporation of HGTi into the chromosome, the observed “cross-over event” between C11 and C12, and the relatively high number of expressed transposases and integrases indicate that the genome of the NI86/21 strain is prone to frequent recombination events. The mosaic-like structure of the HGT elements reflects also such processes and indicates a “gaining and losing” process during their interspecies pilgrimage. The genome expansion of the NI86/21 strain involves a high number of hypothetical proteins, which enormously enriches the genome, potentially providing greater functional redundancy, diversity, and innovation. However, the HGT elements also encode a large number of transposable elements whose expression represents a clear danger for the cells by increasing the number of recombination events.

These findings underscore the exceptional adaptive potential of *R. erythropolis* NI86/21, shaped by extensive horizontal gene transfer, structural genome plasticity, and a vast repertoire of hypothetical proteins with emerging functionality. The combined genomic and proteomic insights presented here not only illuminate the strain’s capacity to thrive in dynamic soil ecosystems but also establish NI86/21 as a valuable model for studying microbial evolution, environmental resilience, and xenobiotic degradation pathways.

## Data Availability

The whole genome sequencing data sets that support the conclusions of this study are accessible through the NCBI repository under BioProject accession number PRJNA625859. This Whole Genome Shotgun project has been deposited at DDBJ/EMBL/GenBank under the accession JABBPH000000000.1.

## SUPPLEMENTAL MATERIAL

Fig. S1 aem.02407-25-s0001.jpg Agarose gel electrophoresis of tDNA extracted from NI86/21.

Table S1 aem.02407-25-s0002.xlsx Summary of annotated genomic and proteomic features of NI86/21.

Table S2 aem.02407-25-s0003.xlsx Mosaic-like structure of HGT islands and extrachromosomal elements of NI86/21.

Table S3 aem.02407-25-s0004.xlsx Integrases, transposases, and recombinases identified in NI86/21.

Table S4 aem.02407-25-s0005.xlsx Enzymes involved in resistance, bioconversion, and degradation.

Table S5 aem.02407-25-s0006.xlsx Proteins of NI86/21 identified by LC-MS/MS in five repetitions.

Table S6 aem.02407-25-s0007.xlsx Ribosomal proteins of NI86/21.

ASM does not own the copyrights to Supplemental Material that may be linked to, or accessed through, an article. The authors have granted ASM a non-exclusive, world-wide license to publish the Supplemental Material files. Please contact the corresponding author directly for reuse.
