# Supplementary figures and images for: Genomic and proteomic analyses of the maize root isolate Rhodococcus erythropolis NI86/21 reveal extensive genome plasticity and parallel evolution of herbicide degradation
Source: Appl Environ Microbiol. 2026 Jan 28;92(2):e02407-25. doi: 10.1128/aem.02407-25 (PMC12915292; doi:10.1128/aem.02407-25)

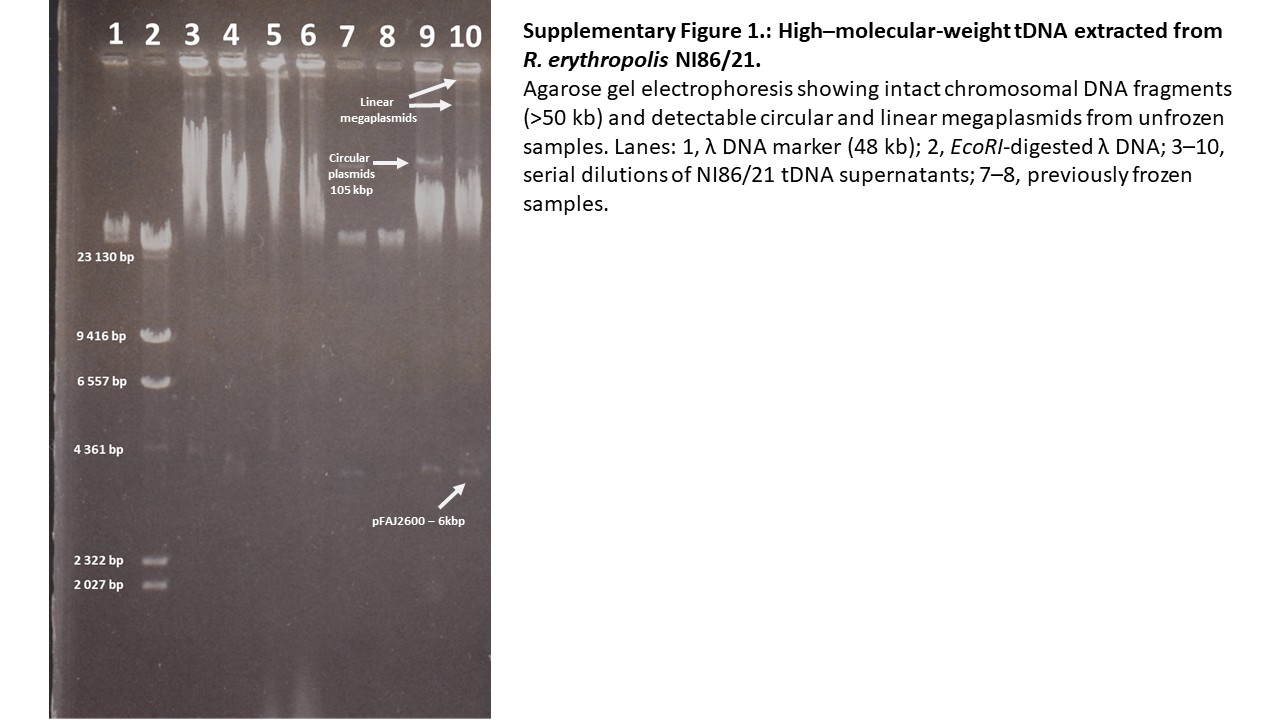

Supplement: Fig. S1 — Agarose gel electrophoresis of tDNA extracted from NI86/21. [file aem.02407-25-s0001.jpg]
